# Emotional-informational support and post-traumatic growth in skin disorders

**DOI:** 10.4102/hsag.v30i0.2981

**Published:** 2025-08-29

**Authors:** Shannon J. Hawker, Ntsoaki F. Tadi, Lehlohonolo Makhakhe

**Affiliations:** 1 Department of Psychology, Faculty of Humanities, University of the Free State, Bloemfontein, South Africa; 2 Department of Dermatology, Faculty of Health Sciences, University of the Free State, Bloemfontein, South Africa

**Keywords:** emotional-informational support, post-traumatic growth, self-esteem, psychophysiological skin disorders, dermatitis, psoriasis, pruritus

## Abstract

**Background:**

Psychophysiological skin disorders’ high prevalence necessitates establishing factors that enable the development of personal strength, self-esteem and receiving adequate support.

**Aim:**

To investigate the relationship between self-esteem and post-traumatic growth (PTG), as well as whether aspects of perceived social support moderate or mediate the relationship between self-esteem and PTG in participants living with psychophysiological skin disorders.

**Setting:**

The study was conducted in South Africa.

**Methods:**

Quantitative, non-experimental, and correlational research design was utilised. 100 participants diagnosed with psychophysiological skin disorders, including atopic dermatitis, psoriasis, pruritus, and alopecia, were selected using a non-probability convenience sampling method. The Post-traumatic Growth Inventory (PTGI) and the Rosenberg Self-Esteem Scale (RSES) were used. Data was analysed using the Statistical Package of the Social Sciences (SPSS) version 28.

**Results:**

Pearson product-moment correlation coefficients and multiple hierarchical regression analyses were performed to investigate the research objectives. A significant positive relationship was found in participants between self-esteem and PTG, with coefficients indicating a moderate to large effect size. As an aspect of perceived social support, emotional-informational support acts as a moderator between self-esteem and PTG. Further, tangible support as an aspect of perceived social support was found not to moderate or mediate the relationship between self-esteem and PTG.

**Conclusion:**

The findings of the current study confirm that only emotional-informational support as an aspect of perceived social support moderates the relationship between self-esteem and PTG among participants living with psychophysiological skin disorders.

**Contribution:**

This insight highlights the need for psychosocial interventions that prioritise emotional and informational support dimensions.

## Introduction

Psychophysiological skin disorders such as psoriasis, atopic dermatitis, alopecia, and acne are recognised as disorders significantly influenced by stress (Chateau, Blackbeard & Aldous 2023; Papadopoulos et al. 1998; Picardi et al. 2003). Living with these conditions may be a challenge for individuals not only in terms of physical ailments, pain, and skin discomfort but also in terms of psychological well-being, as such disorders can unfavourably affect the quality of life and induce post-traumatic distress (Shen et al. 2020; Słomian et al. 2018; Zhai et al. 2014). To date, an extensive body of literature has described the co-occurrence of psychophysiological skin disorders and low self-esteem, loss of efficiency, problematic interpersonal relationships, and stigmatisation (Ahmed, Butler & Reichenberg 2013; Hassanin et al. 2018; Picardi & Abeni 2001; Picardi et al. 2005; Sharma et al. 2018). Subsequently, the possibilities for mitigating psychological stressors and identifying post-traumatic growth (PTG) or intervention are often ignored in this population because many skin disorders are dismissed as insignificant and of minor cosmetic consequence (Chateau et al. 2023; Kantor 2021).

Research on psychophysiological skin disorders has long focused on the pathological aspects concerning self-esteem, perceived social support, and skin research in general (Ahmed et al. 2013; Lyakhovitsky et al. 2017; Sharma et al. 2018). Subsequently, the addition of positive psychology perspectives to treatment modalities for these patients and their feasibility has been essentially overlooked in the context of psychophysiological skin patients (Ghosh & Deb 2017).

Post-traumatic growth refers to positive psychological changes that develop due to attempts to cope with the consequences of highly challenging life circumstances (Tedeschi & Calhoun 1996, 2004; Tedeschi et al. 2018). Furthermore, PTG is associated with adaptive outcomes from various types of traumatic events, including medical conditions (e.g. cancer, HIV, spinal cord injury, diabetes) (Liu et al. 2020; Rzeszutek & Gruszczyńska 2018) and non-medical conditions (e.g. natural disasters, war) (Beaglehole et al. 2018). Individuals with higher PTG experience better psychological well-being (e.g. greater life satisfaction and more positive emotions) and less psychological distress (e.g. fewer depressive and anxiety symptoms) (Beaglehole et al. 2018; Liu et al. 2020; Rzeszutek & Gruszczyńska 2018; Tedeschi et al. 2018; Zhang et al. 2021).

While PTG increases personal strength, when considered within the psychophysiological skin population, it is limiting, which reflects the relative lack of importance attached to the psychological growth of those with skin disorders (Kanji 2019; eds. Walker & Papadopoulos 2005). Owing to the health benefits of PTG (Sztonyk & Formella 2020; Zhai et al. 2014; Zhang et al. 2021), clinicians working with this population could benefit by identifying how to foster PTG to ensure a holistic approach. However, individual differences may predispose some people to more growth than others. As such, social factors such as self-esteem, social support, and self-disclosure may affect affective-cognitive processing when managing emotional distress (Zhang et al. 2021). Tedeschi and Calhoun (2004) suggest that positive associations with self-esteem can aid PTG.

People living with psychophysiological skin disorders experience adverse effects on their self-esteem, especially regarding undesirable psychological changes and coping mechanisms (Ahmed et al. 2013; Picardi et al. 2005). People with skin problems feel embarrassment and distress because their affliction is visible to others, which further impairs beliefs, thoughts, and feelings about the self (Reddy & Chaturvedi 2017; Rezaei & Yazdanpanah 2024; Słomian et al. 2018). Socially, they have also suffered immense neglect and stigmatisation by friends, family members, and even healthcare professionals (Bewley 2017; Hassanin et al. 2018; Shah 2017; eds. Walker & Papadopoulos 2005; Zaidi, Hussain & Sudhakaran 2019).

In addition, studies have suggested that perceived social support is a critical determinant of efficient coping with trauma and resultant positive changes (Ogińska-Bulik 2013). However, perceived social support is complex because it is an intervening variable between an individual’s personal characteristics and developing positive post-traumatic changes (Ogińska-Bulik 2013; Sztonyk & Formella 2020). Studies investigating perceived social support focus primarily on tangible support (providing material goods) and emotional-informational support (validation and empathy) (Beutel et al. 2017). Many studies have revealed that the perceived support that family members and friends provide may protect people from developing psychological distress (Chateau et al. 2023; Poudel et al. 2015). People living with psychophysiological skin disorders typically perceive having limited social support because their disorder is often associated with contagion (Chateau et al. 2023; Shah 2017; eds. Walker & Papadopoulos 2005). Perceptions have also shown that the visibility of a skin disfigurement prevents others from approaching (eds. Walker & Papadopoulos 2005). These stilted reactions of others typically result in spirals of aversive emotional responses and social avoidance (Chateau et al. 2023; Shah 2017).

Taken together, the current study aimed to investigate whether a significant relationship exists between self-esteem and PTG in participants living with psychophysiological skin disorders and whether perceived social support mediates or moderates the relationship between self-esteem and PTG in participants living with such disorders.

## Research methods and design

### Sample

A sample of 100 participants with psychophysiological skin disorders, namely, psoriasis, atopic dermatitis, alopecia, and pruritus, were recruited. All participants were screened and diagnosed by a designated dermatologist. The heterogeneous sample comprised all genders and ethnic groups, with an average age of 39.9 years and a standard deviation of 12.5 years. Participants’ ages ranged from 18 to 60 years. The average number of months they were diagnosed was 39.7 (3 years and 3 months), with a standard deviation of 18.9 months (Table 1). Inclusion criteria included all participants diagnosed with psychophysiological skin disorders within the past 6–72 months. Participants younger than 18 years and older than 60 were excluded from the study, as well as those diagnosed with other skin disorders, such as pathophysiological disorders.

**TABLE 1:** Frequency distributions of biographical variables.

Biographical variables	*n*	%
**Gender**		
Male	39	39.0
Female	61	61.0
**Ethnicities**		
African or Black	72	72.0
Coloured	4	4.0
Indian	1	1.0
White	23	23.0
**Home languages**		
English	13	13.0
Afrikaans	17	17.0
IsiXhosa	12	12.0
IsiZulu	1	1.0
Sesotho	52	52.0
Tshivenda	1	1.0
Setswana	4	4.0
**Types of skin disorder**		
Atopic dermatitis	34	34.0
Psoriasis	25	25.0
Alopecia (clinical sign)	12	12.0
Pruritus (clinical sign)	29	29.0

### Procedure

After their visit by the dermatologist, participants were introduced to the study and, if interested, were sent to the researcher’s office to participate. Once informed written consent was given, they were requested to complete standardised questionnaires assessing self-esteem, social support, and PTG at the hospital during clinic days.

### Measures

This study applied three measures. Firstly, the Post-traumatic Growth Inventory (PTGI) (Tedeschi & Calhoun 1996), comprising 21 items, was used to measure the participants’ positive outcomes after the experience of a traumatic event. The instrument is a widely used measure of PTG and has been reported to have good psychometric properties (Tedeschi & Calhoun 2006).

Secondly, the Brief Social Support Scale (BS6) (Beutel et al. 2017) was used to determine the participants’ perceived social support based on the amount of tangible, emotional, and informational support available. This instrument consists of six items and two subscales (tangible and emotional-informational support, three items each). It has been reported to have good psychometric properties (Beutel et al. 2017).

Thirdly, the Rosenberg Self-Esteem Scale (RSES) was used, a 10-item, well-validated global self-worth scale that measures positive and negative feelings and beliefs about the self (Rosenberg 1965). This scale has shown good internal consistency, stability, and construct validity. The reliability coefficients of the respective measuring instruments in this study were calculated using Cronbach’s-a coefficients and the omega coefficient (Table 2).

**TABLE 2:** Reliability of measuring instruments and subscales.

Measurement scales	α-coefficients	Omega-coefficients
**Post-traumatic growth**		
Factor 1: Relating to others	0.91	0.91
Factor 2: New possibilities	0.89	0.89
Factor 3: Personal strength	0.86	0.87
Factor 4: Spiritual change	0.66	0.66
Factor 5: Appreciation of life	0.85	0.85
**Self-esteem**	0.83	0.83
Perceived social support	-	-
Tangible	0.93	0.93
Emotional-informational	0.88	0.89

### Statistical analysis

The current study used SPSS (IBM 2017) to analyse the results. Pearson product-moment correlation coefficients were used to investigate the first objective of whether a relationship exists between self-esteem and PTG in participants living with psychophysiological skin disorders. Multiple hierarchical regression analyses were performed to investigate the second research objective: specifically, the possible role that perceived social support may play in the relationship between self-esteem and PTG in participants living with psychophysiological skin disorders. The possible role is whether perceived social support mediates or moderates the relationship.

### Ethical considerations

The study was approved by the Ethics Committee of the Faculty of Humanities (GHREC), the Health Sciences Research Ethics Committee at the University of the Free State (UFS-HSD2020/1880/2004), and the Department of Health and complied with the Declaration of Helsinki. The hospital’s Department of Dermatology also granted permission. All participants were informed about how their data would be used for research, and they provided their written consent to participate.

## Results

### Correlation analysis

For this study, Pearson product-moment correlations (Table 3) were calculated to investigate the associations between self-esteem and PTG. As can be seen, the PTG variables show a significantly positive relationship between self-esteem and perceived social support. The self-esteem variable reported significantly positive correlations with the PTG variables (*r* = 0.56, 0.39, 0.48, 0.45; *p* ≤ 0.01). In answer to the first research objective, results show that the higher an individual’s self-esteem, the more likely they are to possess PTG factors. Similarly, the Perceived Social Support (PSS) variables (Tan and Emo) reported significantly positive correlations (1% significance level) with the PTG variables.

**TABLE 3:** Correlations between all the variables for the total group (*N* = 100).

Variables	F1	F2	F3	F5	Ros	Tan	Emo
Factor 1 (F1)	-	0.79*	0.81*	0.73*	0.56*	0.36*	0.56*
Factor 2 (F2)	-	-	0.83*	0.85*	0.39*	0.52*	0.58*
Factor 3 (F3)	-	-	-	0.82*	0.48*	0.40*	0.48*
Factor 5 (F5)	-	-	-	-	0.45*	0.47*	0.49*
Self-esteem (Ros)	-	-	-	-	-	0.15	0.32*
Tangible (Tan)	-	-	-	-	-	-	0.65*
Emotional (Emo)	-	-	-	-	-	-	-

* , *p* ≤ 0.01.

In addition, the correlation between self-esteem and emotional-informational support was of a moderate effect size (*r* = 0. 32, *p* ≤ 0.01). However, the effect size in the correlation between self-esteem and tangible support was non-significant and should be interpreted with caution (*r* = 0.15, *p* ≤ 0.01).

### Multiple hierarchical regression analyses

Multiple hierarchical regression analyses were calculated to determine the possible role (mediator or moderator) of the effect of perceived social support (two scales) on the relationship between self-esteem and PTG (four scales) in participants living with psychophysiological skin disorders. Table 4 depicts the investigation of the role of tangible support (as a perceived social support aspect) in the relationship between self-esteem and PTG (relating to others, new possibilities, personal strength, and appreciation of life) in participants living with psychophysiological skin disorders.

**TABLE 4:** Hierarchical regression analysis predicting post-traumatic growth with self-esteem as an independent variable and tangible (perceived social support) as an intervening variable.

Variables	Step 1	Step 2	Step 3
**Factor 1: Relating to others**			
Self-esteem†	0.55	0.52	0.52
Tangible†	-	0.28	0.29
Self-esteem × Tangible†	-	-	−0.04
Model *R*^2^	0.31	0.39	0.39
Model Δ*R*^2^	0.31	0.08	0.00
**Factor 2: New possibilities**			
Self-esteem†	0.39	0.32	0.32
Tangible†	-	0.47	0.47
Self-esteem × Tangible†	-	-	−0.03
Model *R*^2^	0.15	0.37	0.37
Model Δ*R*^2^	0.15	0.22	0.00
**Factor 3: Personal strength**			
Self-esteem†	0.47	0.42	0.42
Tangible†	-	0.34	0.34
Self-esteem × Tangible†	-	-	0.00
Model *R*^2^	0.23	0.34	0.34
Model Δ*R*^2^	0.23	0.11	0.00
**Factor 4: Appreciation of life**			
Self-esteem†	0.45	0.39	0.39
Tangible†	-	0.41	0.41
Self-esteem × Tangible†	-	-	−0.01
Model *R*^2^	0.21	0.37	0.37
Model Δ*R*^2^	0.21	0.16	0.00

† , Standardised beta coefficients are indicated.

As indicated in Table 4, no statistically significant interaction effect was found (β = −0.043; *t* =−0.533; *p* ≤ 0.595). Therefore, tangible support does not mediate or moderate the relationship between self-esteem and PTG (relating to others) for participants living with psychophysiological skin disorders.

Table 5 depicts the investigation of the role of emotional-informational support (as a social support aspect) in the relationship between self-esteem and PTG (relating to others, new possibilities, personal strength, and appreciation of life) in participants living with psychophysiological skin disorders.

**TABLE 5:** Hierarchical regression analysis predicting post-traumatic growth with self-esteem as an independent variable and emotional-informational support (perceived social support) as intervening variable.

Variables	Step 1	Step 2	Step 3
**Factor 1: Relating to others**			
Self-esteem†	0.56	0.42	0.41
Emotional†		0.43	0.43
Self-esteem × Emotional†			0.10
Model *R*^2^	0.31	0.47	0.48
Model Δ*R*^2^	0.31	0.16	0.01
**Factor 2: New possibilities**			
Self-esteem†	0.39	0.23	0.21
Emotional†		0.51	0.52
Self-esteem × Emotional†			0.11
Model *R*^2^	0.15	0.38	0.40
Model Δ*R*^2^	0.15	0.23	0.01
**Factor 3: Personal strength**			
Self-esteem†	0.47	0.36	0.34
Emotional†		0.36	0.37
Self-esteem × Emotional†			0.13
Model *R*^2^	0.23	0.34	0.36
Model Δ*R*^2^	0.23	0.12	0.02*
**Factor 4: Appreciation of life**			
Self-esteem†	0.45	0.33	0.33
Emotional†		0.38	0.38
Self-esteem × Emotional†			0.06
Model *R*^2^	0.21	0.34	0.34
Model Δ*R*^2^	0.21	0.13	0.00

* , *p* ≤ 0.1.

† , Standardised beta coefficients are indicated.

The result indicated a statistically significant interaction effect at the 10% level (Δ*R*^2^ = 0.016, *F*_1;96_ = 2.463; *p* ≤ 0.010). Therefore, emotional-informational support does indeed moderate the relationship between self-esteem and personal strength in participants living with psychophysiological skin disorders.

The nature of this moderator effect was investigated by calculating the strength and direction of the relationship between self-esteem and personal strength for those patients who achieved low and high scores on the moderator variable (emotional-informational support). For this purpose, two separate regression lines were calculated – one for those high on emotional-informational support (at or above the 75th percentile, *N* = 22; a score of 10 or higher) and one for those low on emotional-informational support (at or lower than the 25th percentile, *N* = 22; a score of 5 or lower).

Participants with high levels of emotional-informational support increase their levels of personal strength with increased self-esteem. A significant positive correlation (*r* = 0.396; *p* ≤ 0.050) was identified for this group between self-esteem and personal growth. However, for the group with low levels of emotional-informational support, a slight decrease in personal strength was identified with increased self-esteem. In this case, no statistically significant relationship (*p* ≤ −0.106; *p* ≤ 638) was identified. Only in the case of participants with high perceived emotional-informational support levels was an increase in self-esteem also noted to increase their personal strength.

## Discussion

This study aimed to identify whether a significant relationship exists between self-esteem and PTG in participants living with psychophysiological skin disorders. As expected, the results indicated that self-esteem positively correlated significantly with PTG. In other words, the higher the level of self-esteem, the higher the development of PTG. This finding resonates with a study by Zhai et al. (2014), which found PTG among participants treated for chronic skin disease, although resilience was the strongest determinant of PTG.

In addition, the study also aimed to identify potentially modifiable factors of perceived social support that play a role in self-esteem and PTG in patients living with psychophysiological skin disorders. As the results indicated, tangible support did not succeed in having any moderating or mediating effects on participants living with psychophysiological skin disorders. This concurs with findings in a study where patients with social anxiety and Type D personalities often needed emotional support, which appeared to be more important than practical, tangible support (Beutel et al. 2017). In contrast, studies have found that tangible support is typically predominant when physical dependency is necessary. Huang and Hsu (2013) reported that tangible support was found to moderate the relationship between depressive symptoms and quality of life in breast cancer patients, which suggests that the more physically disabling the disease, the more tangible the support benefits.

Furthermore, the results revealed a significantly positive relationship between self-esteem, the intervening variable of perceived social support, particularly emotional-informational support (validation and empathy), and PTG. Intrinsically, the higher one’s self-esteem, the more one perceives feeling cared for and validated by one’s close relatives and friends, which will enhance PTG. This finding resonates with Bryson and Bogart’s (2020) study, which found that emotional-informational support is thought to be helped by self-esteem and feelings of personal strength in individuals with rare diseases. Understanding the value of emotional-informational support may contribute significantly to a positive psychology perspective in terms of which interventions could be developed to establish strong social networks to attain PTG among patients with skin disorders. This finding may also increase awareness of the psychological impact of skin disorders, thus increasing collaboration between dermatology and psychology and ensuring a holistic approach to treating patients with skin conditions.

In sum, the results of the multiple hierarchical regression analyses emphasise the practical implications of distinguishing between the emotional-informational and practical, tangible dimensions of perceived social support in participants living with psychophysiological skin disorders.

### Strengths, limitations, and future research

The results of this study cannot be generalised to the larger population because the research study was conducted with a small sample of participants at a hospital in South Africa, limiting the generalisability of the study. However, it is plausible to assume that studies on skin conditions are comparable between different cities in South Africa and that patients are mostly exposed to similar contexts. Nevertheless, further research at more hospitals and community clinics is needed to enhance the meaningfulness of these results. This was a quantitative, correlational study, and causality could not be determined. Notwithstanding that the temporality of association is a strong criterion for causality, cross-sectional studies cannot prove causality. However, they help to generate causal hypotheses. Future studies might consider utilising more advanced statistical methods such as structural equation modelling (SEM) to analyse complex relationships between multiple variables. All variables were measured using self-reporting scales, which may offer an opportunity for the distortion of results given the inherent intentions of the participant. Future studies could adopt a mixed-method approach using qualitative interviews to provide a more in-depth experience of the various variables. Longitudinal research studies are needed to assess the long-term effects of perceived social support on self-esteem and PTG, and this would also be beneficial in measuring and analysing the changes in the variables over time.

The researchers tried to mitigate this effect by being available with an assistant familiar with other languages. The last factor that they took cognisance of was the overlaps in skin conditions that were occasionally experienced by the study participants, which were factored in during data collection. Examples include participants with psoriasis who also had alopecia (hair loss) and atopic and psoriatic participants invariably presenting with pruritus (itching).

Whatever the case, the findings of this study can be utilised to create interventions or psycho-educational programmes in which families and communities may be educated about the aforementioned factors.

### Recommendations

The hospital management has to realise the importance of integrating psychological services into dermatology departments. Emotional-informational support is essential for patients with skin disease, as it addresses their emotional needs, provides valuable health-related information, promotes coping strategies, reduces stigma, enhances treatment adherence, and fosters a sense of belonging within their social network.

## Conclusion

This study emphasises the implications of distinguishing between the emotional-informational and the tangible dimensions of perceived social support in aiding increased self-esteem and PTG among participants living with psychophysiological skin disorders. This significantly highlights the need for a holistic approach to treating people with skin conditions, identifying and understanding the distress patients experience, and the growth factors experienced from having such a condition.

## References

[CIT0001] Ahmed, A., Butler, D.C. & Reichenberg, J., 2013, ‘Quality-of-life effects of common dermatological diseases’, *Seminars in Cutaneous Medicine and Surgery* 32(2), 101–109. 10.12788/j.sder.0009 24049968

[CIT0002] Beaglehole, B., Mulder, R.T., Frampton, C.M., Boden, J.M., Newton-Howes, G. & Bell, C.J., 2018, ‘Psychological distress and psychiatric disorder after natural disasters: Systematic review and meta-analysis’, *British Journal of Psychiatry* 213(6), 716–722. 10.1192/bjp.2018.210 30301477

[CIT0003] Beutel, M.E., Brähler, E., Wiltink, J., Michal, M., Klein, E.M., Jünger, C. et al., 2017, ‘Emotional and tangible social support in a German population-based sample: Development and validation of the brief social support scale (BS6)’, *PLoS One* 12(10), e0186516. 10.1371/journal.pone.0186516 29023540 PMC5638535

[CIT0004] Bewley, A., 2017, ‘The neglected psychological aspects of skin disease’, *British Medical Journal* 358, j3208. 10.1136/bmj.j3208 28684435

[CIT0005] Bryson, B.A. & Bogart, K.R., 2020, ‘Social support, stress, and life satisfaction among adults with rare diseases’. *Health Psychology* 39(10), 912–920. 10.1037/hea0000905 32584069

[CIT0006] Chateau, A.V., Blackbeard, D. & Aldous, C., 2023, ‘The impact of epidermolysis bullosa on the family and healthcare practitioners: A scoping review’, *International Journal of Dermatology* 62(4), 459–475. 10.1111/ijd.16197 35524482

[CIT0007] Ghosh, A. & Deb, A., 2017, ‘Positive psychology interventions for chronic physical illnesses: A systematic review’, *Psychological Studies* 62(3), 213–232. 10.1007/s12646-017-0421-y

[CIT0008] Hassanin, A.M., Ismail, N.N., El Guindi, A. & Sowailam, H.A., 2018, ‘The emotional burden of chronic skin disease dominates physical factors among women, adversely affecting quality of life and sexual function’, *Journal of Psychosomatic Research* 115, 53–57. 10.1016/j.jpsychores.2018.10.011 30470317

[CIT0009] Huang, C.Y. & Hsu, M.C., 2013, ‘Social support as a moderator between depressive symptoms and quality of life outcomes of breast cancer survivors’, *European Journal of Oncology Nursing* 17(6), 767–774. 10.1016/j.ejon.2013.03.011 23623178

[CIT0010] IBM, 2017, *SPSS statistics for Windows, Version 28.0*, IBM Corp, Armonk, NY.

[CIT0011] Kanji, A., 2019, ‘Perspective on living with a skin condition and its psychological impact: A survey’, *Journal of Patient Experience* 6(1), 68–71. 10.1177/2374373518774397 31236454 PMC6572926

[CIT0012] Kantor, J., 2021, ‘Skin disease, sleep, and mental health’, *Journal of the American Academy of Dermatology* 84(1), 36. 10.1016/j.jaad.2020.10.058 33190782

[CIT0013] Liu, Z., Doege, D., Thong, M.S. & Arndt, V., 2020, ‘The relationship between posttraumatic growth and health-related quality of life in adult cancer survivors: A systematic review’, *Journal of Affective Disorders* 276, 159–168. 10.1016/j.jad.2020.07.044 32697695

[CIT0014] Lyakhovitsky, A., Gilboa, S., Eshkol, A., Barzilai, A. & Baum, S., 2017, ‘Late-onset alopecia areata: A retrospective cohort study’, *Dermatology* 233(4), 289–294. 10.1159/000481881 29212074

[CIT0015] Mahoney, J.R., Holtzer, R. & Verghese, J., 2023, ‘Perceived social support dimensions and cognitive outcomes in older adults: A longitudinal study’, *Journal of Aging and Health* 35(3–4), 277–295. 10.1177/08982643241279879

[CIT0016] Ogińska-Bulik, N., 2013, ‘The role of social support in posttraumatic growth in people struggling with cancer’, *Health Psychology Report* 1(1), 1–8. 10.5114/hpr.2013.40464

[CIT0017] Papadopoulos, L., Bor, R., Legg, C. & Hawk, J.L., 1998, ‘Impact of life events on the onset of vitiligo in adults: Preliminary evidence for a psychological dimension in aetiology’, *Clinical and Experimental Dermatology* 23(6), 243–248. 10.1046/j.1365-2230.1998.00384.x 10233617

[CIT0018] Picardi, A. & Abeni, D., 2001, ‘Stressful life events and skin diseases: Disentangling evidence from myth’, *Psychotherapy and Psychosomatics* 70(3), 118–136. 10.1159/000056237 11340413

[CIT0019] Picardi, A., Mazzotti, E., Gaetano, P., Cattaruzza, M.S., Baliva, G., Melchi, C.F. et al., 2005, ‘Stress, social support, emotional regulation, and exacerbation of diffuse plaque psoriasis’, *Psychosomatics* 46(6), 556–564. 10.1176/appi.psy.46.6.556 16288135

[CIT0020] Picardi, A., Pasquini, P., Cattaruzza, M.S., Gaetano, P., Baliva, G., Melchi, C.F. et al., 2003, ‘Only limited support for a role of psychosomatic factors in psoriasis: Results from a case-control study’, *Journal of Psychosomatic Research* 55(3), 189–196. 10.1016/S0022-3999(02)00574-3 12932790

[CIT0021] Poudel, K.C., Buchanan, D.R., Amiya, R.M. & Poudel-Tandukar, K., 2015, ‘Perceived family support and antiretroviral adherence in HIV-positive individuals: Results from a community-based positive living with HIV study’, *International Quarterly of Community Health Education* 36(1), 71–91. 10.1177/0272684X15614220 26525224

[CIT0022] Reddy, M.S. & Chaturvedi, S.K., 2017, ‘Psychosocial issues in dermatology’, *European Medical Journal Dermatology* 5(1), 83–89. 10.33590/emjdermatol/10312653

[CIT0023] Rezaei, N. & Yazdanpanah, N., 2024, ‘Introduction to psychoneuroimmunology and diseases’, in N. Rezaei & N. Yazdanpanah (eds.), *PsychoNeuroImmunology: Volume 2 – Interdisciplinary approaches to diseases*, pp. 1–12, Springer Nature Switzerland AG, Cham.

[CIT0024] Rosenberg, M., 1965, *Society and the adolescent self-image*, Princeton University Press, Princeton, NJ.

[CIT0025] Rzeszutek, M. & Gruszczyńska, E., 2018, ‘Posttraumatic growth among people living with HIV: A systematic review’, *Psychosomatic Research* 114, 81–91. 10.1016/j.jpsychores.2018.09.006 30314584

[CIT0026] Shah, R.B., 2017, ‘Impact of collaboration between psychologists and dermatologists: UK hospital system example’, *International Journal of Women’s Dermatology* 4(1), 8–11. 10.1016/j.ijwd.2017.10.003 PMC5986107 29872670

[CIT0027] Shan, L., Zhang, T., Liu, W. & Zhao, Y., 2025, ‘The relationship between perceived social support and mental workload among clinical nurses: The mediating role of positive coping’, *BMC Nursing* 24(331), a88. 10.1186/s12912-025-02992-3 PMC11948805 40148906

[CIT0028] Sharma, D., Mathur, D.M., Jeenger, J., Sharma, M. & Gupta, K., 2018, ‘Quality of life and self-esteem in patients with psoriasis, vitiligo and healthy controls: Cross sectional study’, *Telangana Journal of Psychiatry* 4(2), 96–102. 10.18231/2455-8559.2018.0011

[CIT0029] Shen, M., Xiao, Y., Su, J., Zhao, S., Li, J., Tao, J. et al., 2020, ‘Prevalence and patient-reported outcomes of noncommunicable skin diseases among college students in China’, *JAAD International* 1(1), 23–30. 10.1016/j.jdin.2020.03.003 34409315 PMC8361873

[CIT0030] Słomian, A., Łakuta, P., Bergler-Czop, B. & Brzezińska-Wcisło, L., 2018, ‘Self-esteem is related to anxiety in psoriasis patients: A case-control study’, *Journal of Psychosomatic Research* 114, 45–49. 10.1016/j.jpsychores.2018.09.005 30314578

[CIT0031] Sztonyk, B.M. & Formella, Z.S., 2020, ‘The role of social support in contributing to posttraumatic growth in persons with vision impairment’, *Health Psychology Report* 8(3), 238–247. 10.5114/hpr.2020.96896

[CIT0032] Tedeschi, R.G. & Calhoun, L.G., 1996, ‘The posttraumatic growth inventory: Measuring the positive legacy of trauma’, *Journal of Trauma and Stress* 9(3), 455–471. 10.1007/BF02103658 8827649

[CIT0033] Tedeschi, R.G. & Calhoun, L.G., 2004, ‘Posttraumatic growth: Conceptual foundations and empirical evidence’, *Psychological Inquiry* 15(1), 1–18. 10.1207/s15327965pli1501_01

[CIT0034] Tedeschi, R.G. & Calhoun, L.G., 2006, *Handbook of posttraumatic growth: Research & practice*, Lawrence Erlbaum Associates, Mahwah, NJ.

[CIT0035] Tedeschi, R.G., Shakespeare-Finch, J., Taku, K. & Calhoun, L.G., 2018, *Posttraumatic growth: Theory, research, and applications*, Routledge, New York, NY.

[CIT0036] Walker, C. & Papadopoulos, L. (eds.), 2005, *Psychodermatology: The psychological impact of skin disorders*, Cambridge University Press, Cambridge.

[CIT0037] Zaidi, Z., Hussain, K. & Sudhakaran, S., 2019, ‘Psychocutaneous disorders’, in *Treatment of skin diseases: A practical guide*, pp. 381–386, Springer, Cham.

[CIT0038] Zhai, J., Huang, Y., Gao, X., Jiang, H. & Xu, J., 2014, ‘Post-trauma growth in a mainland Chinese population with chronic skin disease’, *International Journal of Dermatology* 53(4), 450–457. 10.1111/j.1365-4632.2012.05734.x 24650075

[CIT0039] Zhang, N., Xiang, X., Zhou, S., Liu, H., He, Y. & Chen, J., 2021, ‘Physical activity intervention and posttraumatic growth: A systematic review and meta-analysis’, *Journal of Psychosomatic Research* 152, 110675. 10.1016/j.jpsychores.2021.110675 34823114

